# LncRNA UCA1 elevates the resistance of human leukemia cells to daunorubicin by the PI3K/AKT pathway via sponging miR-613

**DOI:** 10.1042/BSR20201389

**Published:** 2021-06-10

**Authors:** Qiying Yao, Li Zhang, Yuchuan Wang, Junli Liu, Liu Yang, Yingjie Wang

**Affiliations:** 1 College of Basic Medical Sciences, Dalian Medical University, Dalian, Liaoning, China; 2 Department of Pediatrics, The Second Hospital of Dalian Medical University, Dalian, Liaoning, China

**Keywords:** acute leukemia, DNR, miR-613, P13K/AKT, UCA1

## Abstract

Acute leukemia is a hematological malignant tumor. Long non-coding RNA urothelial cancer-associated 1 (UCA1) is involved in the chemo-resistance of diverse cancers, but it is unclear whether UCA1 is associated with the sensitivity of acute leukemia cells to daunorubicin (DNR). DNR (100 nM) was selected for functional analysis. The viability, cell cycle progression, apoptosis, and invasion of treated acute leukemia cells (HL-60 and U-937) were evaluated by cell counting kit-8 (CCK-8) assay, flow cytometry assay, or transwell assay. Protein levels were detected with Western blot analysis. Expression patterns of UCA1 and miR-613 were assessed by quantitative real-time polymerase chain reaction (qRT-PCR). The relationship between UCA1 and microRNA-613 (miR-613) was verified by dual-luciferase reporter assay. We observed that UCA1 expression was elevated in HL-60 and U-937cells. DNR constrained viability, cell cycle progression, invasion, and facilitated apoptosis of HL-60 and U-937 cells in a dose-dependent manner, but these impacts mediated by DNR were reverted after UCA1 overexpression. MiR-613 was down-regulated in HL-60 and U-937 cells, and UCA1 was verified as a miR-613 sponge. MiR-613 inhibitor reversed DNR treatment-mediated effects on viability, cell cycle progression, apoptosis, and invasion of HL-60 and U-937 cells, but these impacts mediated by miR-613 inhibitor were counteracted after UCA1 inhibition. Notably, the inactivation of the PI3K/AKT pathway caused by DNR treatment was reversed after miR-613 inhibitor introduction, but this influence mediated by miR-613 inhibitor was offset after UCA1 knockdown. In conclusion, UCA1 up-regulation facilitated the resistance of acute leukemia cells to DNR via the PI3K/AKT pathway by sponging miR-613.

## Introduction

Acute leukemia is a malignant clonal disease of hematopoietic stem/progenitor cells, which includes acute lymphocytic leukemia (ALL) and acute myeloid leukemia (AML) [1]. The treatment of acute leukemia includes chemotherapy, immunotherapy, radiotherapy, targeted therapy, or stem cell therapy [2]. Although these treatments improve the prognosis of patients with acute leukemia, chemotherapy resistance remains a major problem [3,4].

Long non-coding RNAs (lncRNAs) are a kind of non-protein encoding RNAs that exert crucial regulatory roles in gene regulatory networks [5]. Studies have revealed that lncRNAs are involved in many pathological and physiological processes, such as tumorigenesis, organogenesis, cell lineage choice, and tissue homeostasis [6–9]. Increased researches have revealed that lncRNAs are associated with tumor chemo-resistance [10]. Long non-coding RNA urothelial cancer-associated 1 (UCA1) has been reported to be involved in the occurrence and progression of multiple tumors [11]. Mounting researches have pointed out that UCA1 participates in the chemo-resistance in a series of tumors. For instance, UCA1 exerts a promoting influence on cisplatin resistance in oral squamous cell cancer [12] and bladder cancer [13]. Also, UCA1 up-regulation elevates the resistance of ovarian cancer cells to paclitaxel [14]. Furthermore, UCA1 also facilitates the progression of chronic myeloid leukemia and AML [15,16]. Additionally, UCA1 is associated with Adriamycin resistance in pediatric AML [17]. Nevertheless, the mechanism by which UCA1 regulates daunorubicin (DNR) resistance in acute leukemia remains unclear.

MicroRNAs (miRNAs) are a large class of non-coding RNAs that generally bind to mRNAs to cause translation inhibition or degradation [18]. Researchers have reported that miRNAs play vital roles in intracellular transformation, benign and malignant states, and the progression of cancer [11,19]. MicroRNA-613 (miR-613) has been revealed to play a repressive role in a range of tumors, such as osteosarcoma [20], colorectal cancer [21], and glioma [22]. Moreover, miR-613 increase cisplatin sensitivity in gastric cancer [23]. However, the role of miR-613 in DNR resistance in acute leukemia is unclear.

Consequently, we aimed to survey the function and regulatory mechanism of UCA1 in DNR resistance in acute leukemia.

## Materials and methods

### Cell culture and treatment

Human bone marrow stromal cells HS-5 and two acute leukemia cell lines HL-60 and U-937 were bought from American Type Culture Collection (Manassas, VA, U.S.A.) and cultured in Roswell Park Memorial Institute (RPMI)-1640 medium (Thermo Fisher Scientific, Waltham, MA, U.S.A.) supplemented with fetal bovine serum (FBS, 10%, HyClone, Logan, UT, U.S.A.) and streptomycin/penicillin (1%, HyClone) in an incubator with 5% CO_2_ at 37°C.

For DNR treatment, HL-60 and U-937 cells were cultured in a complete medium supplemented with different doses of DNR (0, 25, 50, and 100 nM) (Sigma, Louis, Missouri, U.S.A.). Moreover, DNR (100 nM)-treated HL-60 and U-937 cells were used for subsequent research.

### Cell transfection

The sequence of UCA1 was cloned into the empty pCDNA3.0 vector (pCDNA3.0-NC) (Invitrogen, Carlsbad, CA, U.S.A.) to construct the overexpression vector of UCA1 (pCDNA3.0-UCA1). Small interference RNA (siRNA) targeting UCA1 (si-UCA1) and its negative control (si-NC) were synthesized by GenePharma (Shanghai, China). MiR-613 mimic (miR-613), scrambled mimic control (miR-NC), miR-613 inhibitor (Anti-miR-613), and scrambled inhibitor control (Anti-miR-NC) were synthesized by GenePharma. Transient transfection was executed using HiPerFect Transfection Reagent (Qiagen, Hilden, Germany). Oligonucleotide sequences were displayed as shown below: si-NC (5′-GCGCGATAGCGCGAATATA-3′), si-UCA1 (5′-GGACAACAGUACACGCAUA-3′), miR-NC (5′-UUCUCCGAACGUGUCACGUTT-3′), miR-613 (5′-AGGAAUGUUCCUUCUUUGCC-3′), Anti-miR-NC (5′-AUCCGUAGGCGUUAGCCUAU-3′), and Anti-miR-613 (5′-GGCAAAGAAGGAACAUUCCT-3′).

### Cell counting kit-8 (CCK-8) assay

The viability of HL-60 and U-937 cells was evaluated by CCK-8 (Dojindo Laboratories, Tokyo, Japan). In short, HL-60 and U-937 cells (1 × 10^4^) were cultured in RPMI-1640 medium supplemented with DNR for 24, 48, and 72 h. Then, the CCK-8 solution (10 μl) was added to each well and incubated for 2 h. The color reaction at 450 nm was measured using Microplate Absorbance Reader (Thermo Fisher Scientific).

### Flow cytometry assay

The apoptosis of HL-60 and U-937 cells with or without DNR treatment was analyzed with the Annexin V-fluorescein isothiocyanate (FITC)/propidium iodide (PI) apoptosis detection kit (BD Biosciences, San Jose, CA, U.S.A.). In brief, HL-60 and U-937 cells were cultured in RPMI-1640 medium supplemented with DNR for 48 h. After washing, HL-60 and U-937 cells (3 × 10^5^) were re-suspended in binding buffer (100 μl). Then, the cells were stained with Annexin V-FITC (5 μl) and PI (100 μl) and incubated for 30 min in the dark. In the end, the cells were analyzed using the FACScan flow cytometry (BD Biosciences).

### Transwell assay

The invasion ability of HL-60 and U-937 cells with or without DNR treatment was assessed by transwell assay. In brief, the cell medium containing HL-60 and U-937 cells (1 × 10^5^) were added to the top chamber (8 μm, BD Biosciences) coated with matrigel matrix (BD Biosciences). Contemporaneously, RPMI-1640 medium with FBS (10%) were added to the down chamber. After culture for 24 h, the cells in the lower chamber were counted by the CCK-8 assay.

### Western blot analysis

Collected HL-60 and U-937 cells were lysed in RIPA lysis buffer (Thermo Fisher Scientific) and then centrifuged for 10 min (13,000 × ***g***, 4°C). Subsequently, the supernatants were collected and quantified with a BCA Protein Assay Kit (Pierce, Holmdel, NJ, U.S.A.). About 30 μg total protein was separated by sodium dodecyl sulphate-polyacrylamide gel electrophoresis (10%, SDS-PAGE). Afterward, the isolated proteins were transferred onto polyvinylidene difluoride (PVDF) membranes (Millipore, Bedford, MA, U.S.A.) and then blocked with Tris Buffered Saline Tween (TBST) buffer with 5% skim milk. The PVDF membranes were then incubated with primary antibodies: anti-cyclin D1 (1:200, ab16663), anti-Vimentin (1:1000, ab137321), anti-Cleaved-caspase-3 (Cleaved-casp-3) (1:500, ab32042), anti-phosphatidylinositol 3-kinase (PI3K) (1:1000, ab32089), anti-phosphorylated PI3K (p-PI3K) (1:500, ab278545) anti-protein kinase B (AKT) (1:10,000, ab179463), anti-phosphorylated-AKT (p-AKT) (1:1000, ab192623), and anti-glyceraldehyde-3-phosphate dehydrogenase (GAPDH) (1:2500, ab9485). Next, the membranes were incubated with goat anti-rabbit or mouse IgG. GAPDH was regarded as a loading control. All antibodies used in the present study were obtained from Abcam (Cambridge, MA, U.S.A.). The bands were visualized through the ImageJ software of National Institutes of Health (Bethesda, MD, U.S.A.).

### Quantitative real-time polymerase chain reaction (qRT-PCR)

Total RNA of HL-60 and U-937 cells was extracted through TRIzol reagent (Thermo Fisher Scientific). About 1 μg total RNA was reverse transcribed into complementary DNA using Moloney Murine Leukemia Virus (M-MLV) First Strand Kit (Life Technologies, Grand Island, NY, U.S.A.) or MiRNA Reverse Transcription kit (Life Technologies). qPCR was executed using 0.1 μg complementary DNA and the SYBR Fast qPCR Mix (Thermo Fisher Scientific). The primer sequences were presented as below: UCA1: 5′-TCGGGTAACTCTTACGGT-3′ (F) and 5′-GGTCCATTGAGGCTGTAG-3′ (R); GAPDH: 5′-GATTCCACCCATGGCAAATTCC-3′ (F) and 5′-TCGCTCCTGGAAGATGGTGAT-3′ (R); miR-613: 5′-GCGCGAGGAATGTTCCTTC-3′ (F) and 5′-AGTGCAGGGTCCGAGGTATT-3′ (R) as well as U6 small nuclear RNA (snRNA): 5′-CTCGCTTCGGCAGCACA-3′ (F) and 5′-AACGCTTCACGAATTTGCGT-3′ (R). GAPDH or U6 snRNA was used as an internal control for UCA1 and miR-613. The levels of UCA1 and miR-613 were calculated by the 2^−ΔΔCt^ method.

### Dual-luciferase reporter assay

The potential binding sites between UCA1 and miR-613 were predicted by the starbase, LncBase and mircode databases. The sequences of wild-type (WT) UCA1 (with predicted miR-613 binding sites) and mutant (MUT) UCA1 were synthesized and inserted into the pGL3-control vector (Promega, Madison, WI, U.S.A.). After that, A luciferase reporter vector were cotransfected into HL-60 and U-937 cells together with miR-NC or miR-613. A dual-luciferase reporter assay kit (Promega) was employed to analyze the luciferase activities.

### Statistical analysis

Data derived from at least three independent experiments were presented as mean ± standard deviation. GraphPad Prism 6.0 (GraphPad, San Diego, CA, U.S.A.) and SPSS 18.0 software (SPSS, Chicago, IL, U.S.A.) were utilized for statistical analysis. *P*<0.05 was deemed to indicate a statistically significant difference. The differences between two or among more groups were analyzed through Student's *t* test or one-way analysis of variance (ANOVA).

## Results

### DNR repressed viability, cell cycle progression, invasion, and triggered apoptosis of HL-60 and U-937 cells

**Figure 1:**
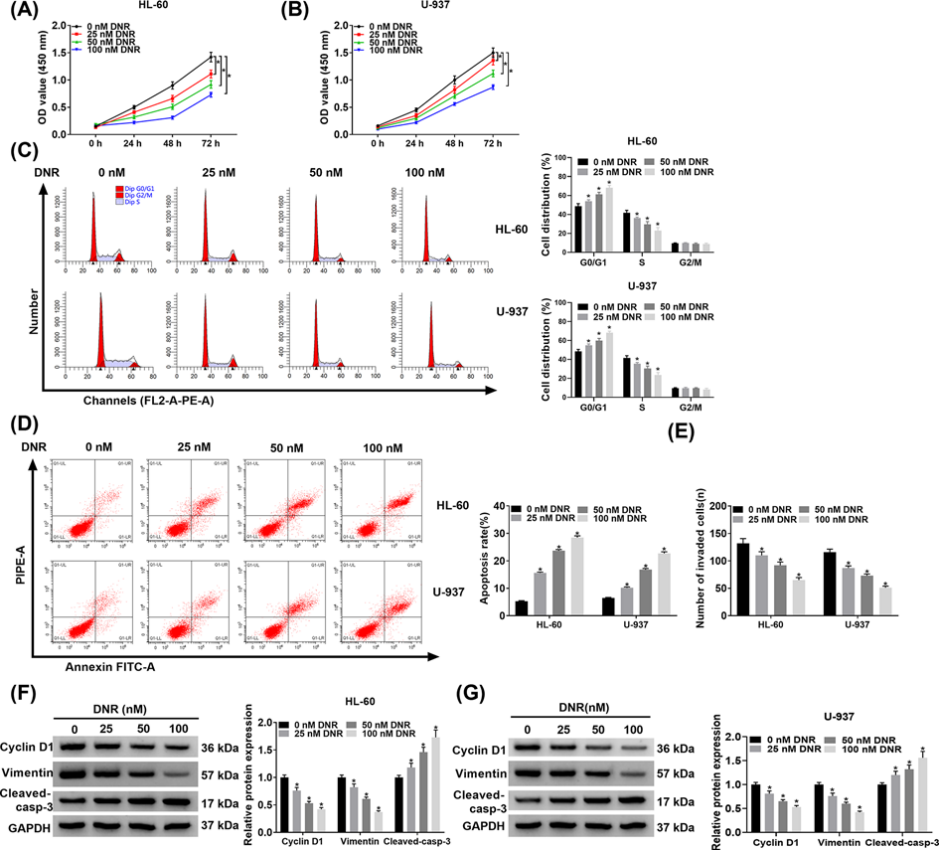
Effects of DNR treatment on malignant behaviors of acute leukemia cells (**A**–**F**) HL-60 and U-937 cells were treated with different DNR doses (0, 25, 50, and 100 nM). (A and B) CCK-8 assay was carried out to analyze the viability of HL-60 and U-937 cells. (C and D) Flow cytometry assay was conducted to determine cell cycle progression and apoptosis of HL-60 and U-937 cells. (E) Transwell assay was employed to evaluate the invasion of HL-60 and U-937 cells. (**F** and **G**) The levels of Cyclin D1, Vimentin, and Cleaved-casp-3 in HL-60 and U-937 cells were detected through Western blot analysis; *P<0.05.

To survey the effects of DNR on malignant behaviors of HL-60 and U-937 cells, we first performed CCK-8 assay to assess the viability of HL-60 and U-937 cells treated with different DNR doses (0, 25, 50, and 100 nM). Compared with the control group, DNR treatment suppressed the viability of HL-60 and U-937 cells with the enhancement of DNR dose (Figure 1A,B). Flow cytometry assay manifested that DNR treatment promoted cell cycle arrest and apoptosis of HL-60 and U-937 cells with the increase of DNR (Figure 1C,D). Transwell assay was then conducted and the results presented that DNR treatment caused a decrease in the invasion ability of HL-60 and U-937 cells in a dose-dependent manner (Figure 1E). Afterward, the levels of Cyclin D1 (proliferation-associated protein), Vimentin (migration-associated protein), and Cleaved-casp-3 (apoptosis-rated apoptosis) were detected and the results exhibited that DNR treatment decreased protein levels of Cyclin D1 and Vimentin in HL-60 and U-937 cells in a dose-dependent manner, while elevated the protein level of Cleaved-casp-3 (Figure 1F,G). Collectively, these results suggested that DNR repressed viability, cell cycle progression, invasion, and induced apoptosis of HL-60 and U-937 cells in a dose-dependent manner.

### DNR treatment decreased UCA1 expression in HL-60 and U-937 cells

**Figure 2:**
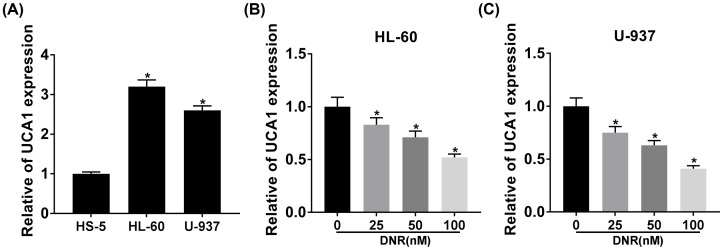
DNR treatment decreased UCA1 expression in acute leukemia cells (**A**) QRT-PCR analysis of UCA1 in HL-60, U-937, and HS-5 cells. (**B** and** C**) The expression of UCA1 in HL-60 and U-937 cells treated with different DNR doses (0, 25, 50, and 100 nM) was analyzed by qRT-PCR; *P<0.05.

To verify the expression of UCA1 in HL-60 and U-937 cells, we executed qRT-PCR analysis to detect UCA1 expression in HL-60 and U-937 cells. We discovered that UCA1 expression was markedly elevated in HL-60 and U-937 cells in comparison with the HS-5 cells (Figure 2A). However, DNR treatment resulted in a decrease in UCA1 expression in HL-60 and U-937 cells (Figure 2B,C). Taken together, these data indicated that DNR treatment reduced UCA1 expression in HL-60 and U-937 cells in a dose-dependent manner.

### UCA1 up-regulation decreased the sensitivity of HL-60 and U-937 cells to DNR

**Figure 3:**
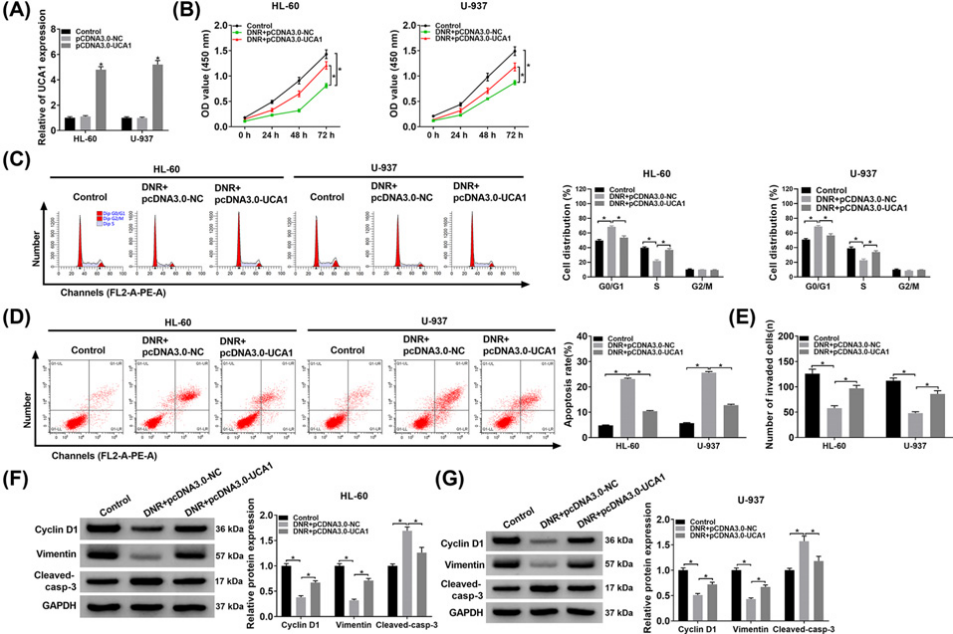
Effect of UCA1 on the sensitivity of acute leukemia cells to DNR (**A**) The expression of UCA1 in HL-60 and U-937 cells transfected with pCDNA3.0-NC or pCDNA3.0-UCA1 was assessed by qRT-PCR. (**B**–**F**) HL-60 and U-937 cells were transfected with pCDNA3.0-NC or pCDNA3.0-UCA1 and then treated with DNR (100 nM). (B) The viability of HL-60 and U-937 cells was determined via CCK-8 assy. (**C** and** D**) The cell cycle progression and apoptosis of HL-60 and U-937 cells was detected through flow cytometry assay. (**E**) The invasion of HL-60 and U-937 cells was analyzed using transwell assay. (**F** and** G**) Protein levels of Cyclin D1, Vimentin, and Cleaved-casp-3 protein in HL-60 and U-937 cells were measured by Western blot analysis; *P<0.05.

Taking into account the above results, we further explored the effects of UCA1 on the viability, apoptosis and invasion of DNR (100 nM)-treated HL-60 and U-937 cells. The overexpression efficiency of pCDNA3.0-UCA1 in HL-60 and U-937 cells was presented in Figure 3A. Moreover, UCA1 overexpression partly reversed the repressive influence of DNR treatment on the viability of HL-60 and U-937 cells (Figure 3B). Moreover, forced UCA1 expression partially abolished the promoting influence of DNR treatment on cell cycle arrest and apoptosis of HL-60 and U-937 cells (Figure 3C,D). Also, transwell assay displayed that UCA1 overexpression reversed the decrease of the invasion ability of HL-60 and U-937 cells caused by DNR treatment (Figure 3E). Additionally, Western blot analysis manifested that UCA1 up-regulation overturned DNR treatment-mediated effects on protein levels of Cyclin D1, Vimentin, and Cleaved-casp-3 in HL-60 and U-937 cells (Figure 3F,G). Therefore, these data indicated that UCA1 up-regulation reduced the sensitivity of HL-60 and U-937 cells to DNR.

### UCA1 served as a sponge of miR-613 in HL-60 and U-937 cells

**Figure 4:**
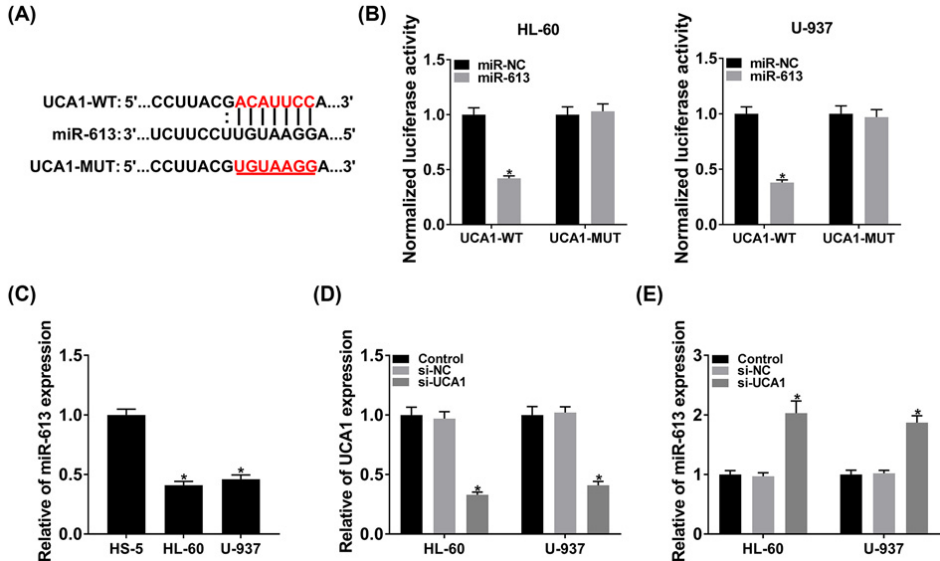
UCA1 acted as a sponge for miR-613 (**A**) The potential binding sites between UCA1 and miR-613. (**B**) Dual-luciferase reporter assay was carried out to assess the luciferase activities of the UCA1-WT and UCA1-MUT reporters in HL-60 and U-937 cells transfected with miR-613 mimic or miR-NC. (**C**) The expression of miR-613 in HS-5, HL-60, and U-937 cells was analyzed using qRT-PCR. (**D**) The knockdown efficiency of si-UCA1 in HL-60 and U-937 cells was validated by qRT-PCR. (**E**) QRT-PCR was executed to analyze the effect of UCA1 knockdown on miR-613 expression in HL-60 and U-937 cells; *P<0.05.

Further experiments revealed that UCA1 was mostly distributed in the cytoplasm of HL-60 and U-937 cells, indicating that UCA1 might act as a miRNA sponge (Supplementary Figure S1). To explore the regulatory mechanism of UCA1 in HL-60 and U-937 cells, we predicted miRNAs that might interact with UCA1. We discovered that four miRNAs (miR-206, miR-613, miR-4770, and miR-4735-3p) had complementary sites with UCA1 in the overlapping starbase, LncBase and mircode databases (Supplementary Figure S2A). Moreover, miR-613 mimic reduced the luciferase activity of the UCA1-WT reporter in both HL-60 and U-937 cells (Supplementary Figure S2B). The overexpression efficiency of miR-613 mimic was exhibited in Supplementary Figure S3. The binding sties between miR-613 and UCA1 were presented in Figure 4A. Dual-luciferase reporter assay exhibited that miR-613 mimic curbed the luciferase activity of the UCA1-WT reporter in HL-60 and U-937 cells, whereas the luciferase activity of the UCA1-MUT reporter did not change (Figure 4B). Compared with the HS-5 cells, the expression of miR-613 was markedly down-regulated in HL-60 and U-937 cells (Figure 4C). QRT-PCR displayed that UCA1 expression was observably reduced in HL-60 and U-937 cells after transfection with si-UCA1 (Figure 4D). In addition, silenced UCA1 expression distinctly elevated the expression of miR-613 in HL-60 and U-937 cells (Figure 4E). In sum, these results demonstrated that UCA1 severed as a sponge for miR-613 expression in HL-60 and U-937 cells.

### UCA1 decreased the sensitivity of HL-60 and U-937 cells to DNR by interacting with miR-613

**Figure 5:**
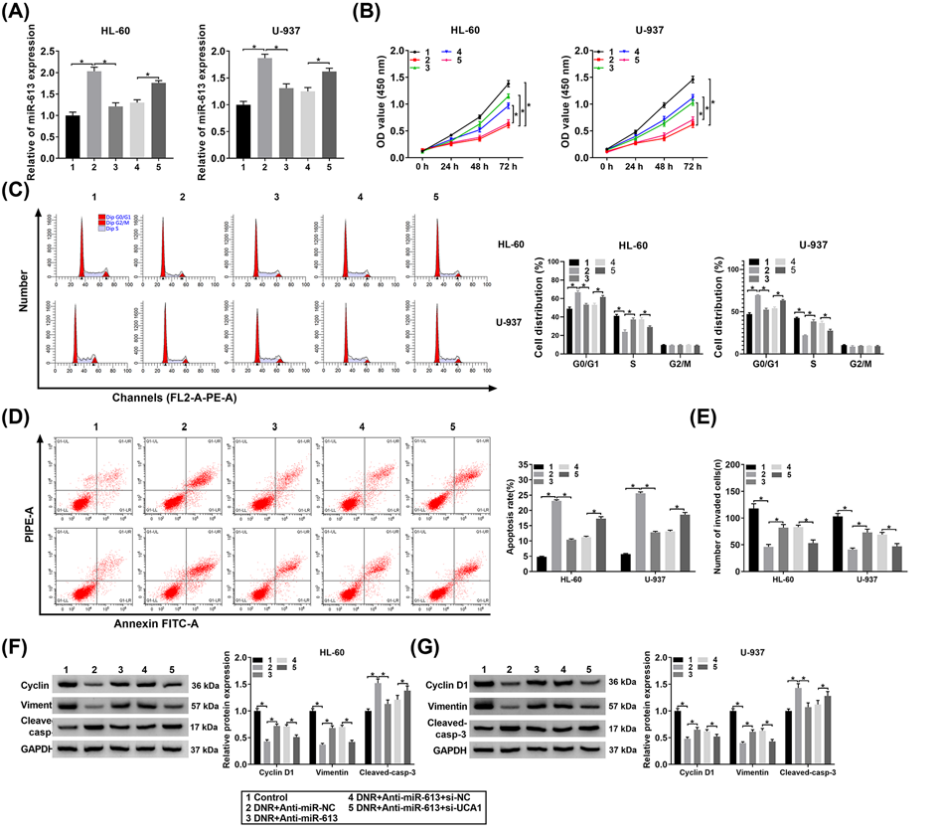
UCA1 interacted with miR-613 to affect acute leukemia cell sensitivity to DNR (**A**–**F**) HL-60 and U-937 cells were transfected with Anti-miR-NC, Anti-miR-613, Anti-miR-613+si-NC, or Anti-miR-613+si-UCA1 and then treated with DNR (100 nM). (A) The expression of miR-613 in HL-60 and U-937 cells was evaluated by qRT-PCR. (B) CCK-8 assay was carried out to determine the viability of HL-60 and U-937 cells. (C and D) The cell cycle progression and apoptosis of HL-60 and U-937 cells was determined with flow cytometry assay. (E) The invasion of HL-60 and U-937 cells was assessed via transwell assay. (**F** and** G**) Western blot analysis was performed to detect protein levels of Cyclin D1, Vimentin, and Cleaved-casp-3 in HL-60 and U-937 cells; *P<0.05.

Given that UCA1 acted as a sponge for miR-613, we further investigated whether UCA1 affected the sensitivity of HL-60 and U-937 cells to DNR via interacting with miR-613. The knockdown efficiency of miR-613 inhibitor was displayed in Supplementary Figure S3. We observed that miR-613 was up-regulated in DNR-treated HL-60 and U-937 cells, while this increase was recovered after Anti-miR-613 introduction (Figure 5A). Moreover, the down-regulation of miR-613 in DNR-treated HL-60 and U-937 cells mediated by miR-613 inhibitor was reversed by UCA1 silencing (Figure 5A). The viability of HL-60 and U-937 cells was elevated by miR-613 inhibition under DNR treatment, while this elevation was abolished by the knockdown of UCA1 (Figure 5B). Furthermore, miR-613 inhibitor promoted cell cycle progression and repressed cell apoptosis in DNR-treated HL-60 and U-937 cells, while these effects caused by miR-613 inhibitor were reversed after UCA1 knockdown (Figure 5C,D). In addition, UCA1 silencing abrogated the promoting impact of miR-613 knockdown on the invasion of DNR-treated HL-60 and U-937 cells (Figure 5E). Also, silenced miR-613 expression increased protein levels of Cyclin D1 and Vimentin and decreased the protein level of Cleaved-casp-3 in DNR-treated HL-60 and U-937 cells, but these trends were reversed by UCA1 inhibition (Figure 5F,G). In short, these results manifested that UCA1 affected the sensitivity of HL-60 and U-937 cells to DNR through miR-613.

### UCA1 regulated the PI3K/AKT pathway via miR-613

**Figure 6:**
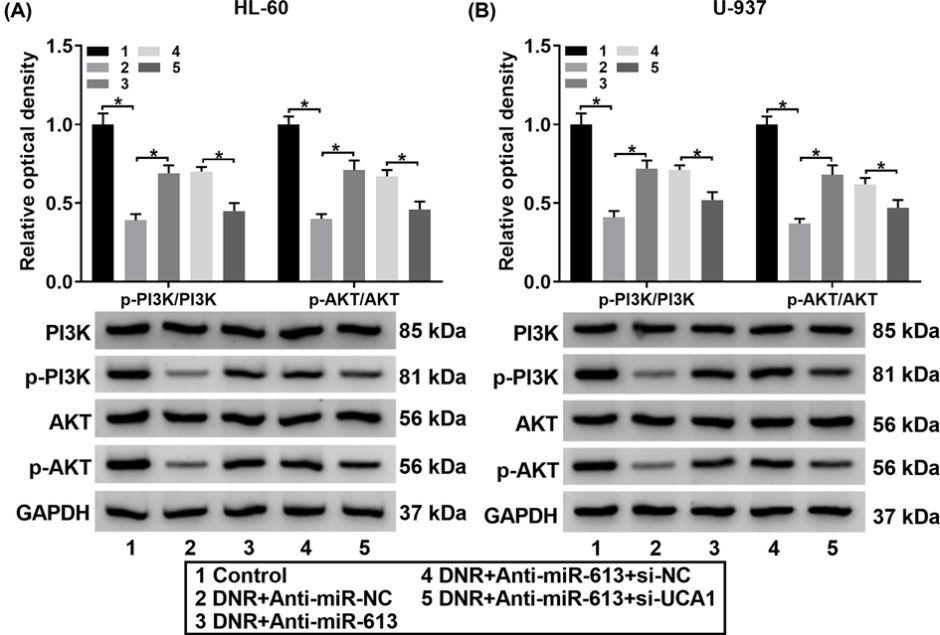
UCA1 mediated the PI3K/AKT pathway via miR-613 (**A** and** B**) Protein levels of PI3K, p-PI3K, AKT, and p-AKT in HL-60 and U-937 cells transfected with Anti-miR-NC, Anti-miR-613, Anti-miR-613+si-NC, or Anti-miR-613+si-UCA1 under DNR treatment were detected through Western blot analysis; *P<0.05.

To further survey the regulatory mechanism of UCA1 in HL-60 and U-937 cells, we assessed the levels of p-PI3K and p-AKT in DNR-treated HL-60 and U-937 cells transfected Anti-miR-NC, Anti-miR-613, Anti-miR-613+si-NC, or Anti-miR-613+si-UCA1. The results exhibited that DNR reduced the value of p-PI3K/P13K and p-AKT/AKT in HL-60 and U-937 cells, while this decrease was reversed by miR-613 silencing (Figure 6A,B). However, the effects of miR-613 knockdown on the value of p-PI3K/PI3K and p-AKT/AKT in DNR-treated HL-60 and U-937 cells were counteracted by UCA1 silencing (Figure 6A,B). Therefore, these data indicated that UCA1 regulated the PI3K/AKT pathway via adsorbing miR-613.

## Discussion

DNR is a chemotherapeutic drug widely utilized in the treatment of various malignant tumors. A number of studies have proved that DNR delayed the growth of cancer cells. Also, the synergy of DNR and celecoxib promotes apoptosis and reduces growth of AML cells [24]. At present, the resistance of acute leukemia cells to DNR is one of the main obstacles to its treatment [25].

Accumulated studies have proved that UCA1 is connected with the chemo-resistance of various cancers. Report of Bian et al. claimed that UCA1 reduced the sensitivity of colorectal cancer cells to 5-fluorouracil and promoted the proliferation of colorectal cancer cells [26]. Another report pointed out that UCA1 elevated doxorubicin resistance in gastric cancer [27]. Xiao et al. stated that UCA1 accelerated the resistance of chronic myeloid leukemia cells to imatinib [15]. Additionally, UCA1 silencing could elevate the cytotoxic effect of adriamycin to adriamycin-treated pediatric AML cells [28]. In the present study, DNR induced apoptosis and impeded viability, cell cycle progression, and invasion of HL-60 and U-937 cells in a dose-dependent manner. Also, DNR restrained the expression of UCA1 in HL-60 and U-937 cells, and forced UCA1 reversed DNR treatment-mediated impacts on viability, cell cycle progression, invasion, and apoptosis of HL-60 and U-937 cells. Thus, we concluded that UCA1 upregulation could reduce the sensitivity of HL-60 and U-937 cells to DNR.

Recent researches have pointed out that UCA1 act as a miRNA sponge and participate in the regulation of chemo-resistance in diversified cancers [14,28,29]. MiR-613 had been reported as an anti-tumor gene in bladder cancer [30], non-small cell lung cancer [31], and laryngeal squamous cell cancer [32]. Moreover, miR-613 mimic elevated cell sensitivity to cisplatin by down-regulating SOX9 in gastric cancer [23]. Herein, miR-613 was down-regulated in HL-60 and U-937 cells. Also, UCA1 was identified as a miR-613 sponge. Furthermore, DNR treatment restored the down-regulation of miR-613 in HL-60 and U-937 cells. In addition, the effects of DNR treatment on viability, cell cycle progression, apoptosis, and invasion of HL-60 and U-937 cells were reversed by miR-613 inhibition, but these effects caused by miR-613 silencing were restored after UCA1 silencing. However, miR-613 was revealed as an oncogene in colon cancer [33] and cervical cancer [34], which might be related to tissues specificity. Thus, we inferred that UCA1 mediated the sensitivity of HL-60 and U-937 cells to DNR via miR-613.

It was reported that the Pi3K/AKT pathway was involved in the chemo-resistance of diverse cancers [35,36]. Yang et al. reported that lncRNA linc00239 contributed to malignant behaviors of AML cells and elevated doxorubicin resistance through activating the PI3K/AKT/mTOR pathway in AML [37]. In the present study, DNR treatment blocked the PI3K/AKT pathway, but miR-613 silencing reversed the inactivation of the PI3K/AKT pathway in HL-60 and U-937 cells caused by DNR treatment. Nevertheless, UCA1 suppression offset the influence of miR-613 inhibitor on the PI3K/AKT pathway in DNR-treated HL-60 and U-937 cells. Therefore, we inferred that UCA1 regulated the sensitivity of HL-60 and U-937 cells to DNR through mediating PI3K/AKT pathway via sponging miR-613.

In sum, UCA1 up-regulation decreased the sensitivity of acute leukemia cells to DNR. Moreover, UCA1 overexpression activated the PI3K/AKT pathway via sponging miR-613. The present study provided an evidence to support UCA1 as a promising target for acute leukemia treatment.

## Supplementary Material

Supplementary Figures S1-S3

## Data Availability

The datasets used and/or analyzed during the current study are available from the corresponding author on reasonable request.

## Abbreviations

AKT: protein kinase B
AML: acute myeloid leukemia
CCK-8: cell counting kit-8
cleaved-casp-3: cleaved-caspase-3
DNR: daunorubicin
miR-613: microRNA-613
p-AKT: phosphorylated-AKT
p-PI3K: phosphorylated PI3K
PI3K: phosphatidylinositol 3-kinase
qRT-PCR: quantitative real-time polymerase chain reaction
UCA1: urothelial cancer-associated 1

## References

[1] Juliusson G. and Hough R. (2016) Leukemia. Progr. Tumor Res. 43, 87–100 10.1159/000447076 27595359

[2] Kantarjian H.M., Keating M.J. and Freireich E.J. (2018) Toward the potential cure of leukemias in the next decade. Cancer 124, 4301–4313 10.1002/cncr.31669 30291792

[3] Gutierrez A. and Kentsis A. (2018) Acute myeloid/T-lymphoblastic leukaemia (AMTL): a distinct category of acute leukaemias with common pathogenesis in need of improved therapy. Br. J. Haematol. 180, 919–924 10.1111/bjh.15129 29441563 PMC5837942

[4] Liu B., Ma X., Liu Q., Xiao Y., Pan S. and Jia L. (2018) Aberrant mannosylation profile and FTX/miR-342/ALG3-axis contribute to development of drug resistance in acute myeloid leukemia. Cell Death Dis. 9, 688 10.1038/s41419-018-0706-7 29880818 PMC5992136

[5] Gibb E.A., Brown C.J. and Lam W.L. (2011) The functional role of long non-coding RNA in human carcinomas. Mol. Cancer 10, 38 10.1186/1476-4598-10-38 21489289 PMC3098824

[6] Schmitz S.U., Grote P. and Herrmann B.G. (2016) Mechanisms of long noncoding RNA function in development and disease. Cell. Mol. Life Sci. 73, 2491–2509 10.1007/s00018-016-2174-5 27007508 PMC4894931

[7] Prensner J.R. and Chinnaiyan A.M. (2011) The emergence of lncRNAs in cancer biology. Cancer Discov. 1, 391–407 10.1158/2159-8290.CD-11-0209 22096659 PMC3215093

[8] Chen M., Zhuang C., Liu Y., Li J., Dai F., Xia M. et al. (2016) Tetracycline-inducible shRNA targeting antisense long non-coding RNA HIF1A-AS2 represses the malignant phenotypes of bladder cancer. Cancer Lett. 376, 155–164 10.1016/j.canlet.2016.03.037 27018306

[9] Chen M., Zhang R., Lu L., Du J., Chen C., Ding K. et al. (2020) LncRNA PVT1 accelerates malignant phenotypes of bladder cancer cells by modulating miR-194-5p/BCLAF1 axis as a ceRNA. Aging 12, 22291–22312 10.18632/aging.202203 33188158 PMC7695393

[10] Ayers D. and Vandesompele J. (2017) Influence of microRNAs and long non-coding RNAs in cancer chemoresistance. Genes (Basel) 8, 95 10.3390/genes8030095 28273813 PMC5368699

[11] Sun Z., Niu S., Xu F., Zhao W., Ma R. and Chen M. (2020) CircAMOTL1 promotes tumorigenesis through miR-526b/SIK2 axis in cervical cancer. Front. Cell Development. Biol. 8, 568190 10.3389/fcell.2020.568190 33344445 PMC7744824

[12] Fang Z., Zhao J., Xie W., Sun Q., Wang H. and Qiao B. (2017) LncRNA UCA1 promotes proliferation and cisplatin resistance of oral squamous cell carcinoma by sunppressing miR-184 expression. Cancer Med. 6, 2897–2908 10.1002/cam4.1253 29125238 PMC5727307

[13] Fan Y., Shen B., Tan M., Mu X., Qin Y., Zhang F. et al. (2014) Long non-coding RNA UCA1 increases chemoresistance of bladder cancer cells by regulating Wnt signaling. FEBS J. 281, 1750–1758 10.1111/febs.12737 24495014

[14] Wang J., Ye C., Liu J. and Hu Y. (2018) UCA1 confers paclitaxel resistance to ovarian cancer through miR-129/ABCB1 axis. Biochem. Biophys. Res. Commun. 501, 1034–1040 10.1016/j.bbrc.2018.05.104 29777711

[15] Xiao Y., Jiao C., Lin Y., Chen M., Zhang J., Wang J. et al. (2017) LncRNA UCA1 contributes to imatinib resistance by acting as a ceRNA against miR-16 in chronic myeloid leukemia cells. DNA Cell Biol. 36, 18–25 10.1089/dna.2016.3533 27854515

[16] Li J., Wang M. and Chen X. (2020) Long non-coding RNA UCA1 modulates cell proliferation and apoptosis by regulating miR-296-3p/Myc axis in acute myeloid leukemia. Cell Cycle 19, 1454–1465 10.1080/15384101.2020.1750814 32286143 PMC7469675

[17] Zhang Y., Liu Y. and Xu X. (2018) Knockdown of LncRNA-UCA1 suppresses chemoresistance of pediatric AML by inhibiting glycolysis through the microRNA-125a/hexokinase 2 pathway. J. Cell. Biochem. 119, 6296–6308 10.1002/jcb.26899 29663500

[18] Bartel D.P. (2004) MicroRNAs: genomics, biogenesis, mechanism, and function. Cell 116, 281–297 10.1016/S0092-8674(04)00045-5 14744438

[19] Mishra S., Yadav T. and Rani V. (2016) Exploring miRNA based approaches in cancer diagnostics and therapeutics. Crit. Rev. Oncol. Hematol. 98, 12–23 10.1016/j.critrevonc.2015.10.003 26481951

[20] Li X., Sun X., Wu J. and Li Z. (2016) MicroRNA-613 suppresses proliferation, migration and invasion of osteosarcoma by targeting c-MET. Am J Cancer Res. 6, 2869–2879 28042506 PMC5199760

[21] Yan H.L., Li L., Li S.J., Zhang H.S. and Xu W. (2016) miR-346 promotes migration and invasion of nasopharyngeal carcinoma cells via targeting BRMS1. J. Biochem. Mol. Toxicol. 30, 602–607 10.1002/jbt.21827 27501413

[22] Sang Q., Liu X. and Sun D. (2018) Role of miR-613 as a tumor suppressor in glioma cells by targeting SOX9. Onco Targets Ther. 11, 2429–2438 10.2147/OTT.S156608 29765228 PMC5942171

[23] Xue M., Li G., Sun P., Zhang D., Fang X. and Li W. (2019) MicroRNA-613 induces the sensitivity of gastric cancer cells to cisplatin through targeting SOX9 expression. Am. J. Transl. Res. 11, 885–894 30899388 PMC6413272

[24] Chen C., Xu W. and Wang C.M. (2013) Combination of celecoxib and doxorubicin increases growth inhibition and apoptosis in acute myeloid leukemia cells. Leuk. Lymphoma 54, 2517–2522 10.3109/10428194.2013.781170 23452119

[25] Hackl H., Astanina K. and Wieser R. (2017) Molecular and genetic alterations associated with therapy resistance and relapse of acute myeloid leukemia, J Hematol Oncol. 10, 51 10.1186/s13045-017-0416-0 28219393 PMC5322789

[26] Bian Z., Jin L., Zhang J., Yin Y., Quan C., Hu Y. et al. (2016) LncRNA-UCA1 enhances cell proliferation and 5-fluorouracil resistance in colorectal cancer by inhibiting miR-204-5p. Sci. Rep. 6, 23892 10.1038/srep23892 27046651 PMC4820696

[27] Shang C., Guo Y., Zhang J. and Huang B. (2016) Silence of long noncoding RNA UCA1 inhibits malignant proliferation and chemotherapy resistance to adriamycin in gastric cancer. Cancer Chemother. Pharmacol. 77, 1061–1067 10.1007/s00280-016-3029-3 27056384

[28] Zhang Y., Liu Y. and Xu X. (2018) Knockdown of LncRNA-UCA1 suppresses chemoresistance of pediatric AML by inhibiting glycolysis through the microRNA-125a/hexokinase 2 pathway. J. Cell. Biochem. 119, 6296–6308 10.1002/jcb.26899 29663500

[29] Li Z., Niu H., Qin Q., Yang S., Wang Q., Yu C. et al. (2019) LncRNA UCA1 mediates resistance to cisplatin by regulating the miR-143/FOSL2-Signaling pathway in ovarian cancer. Mol. Ther. Nucleic Acids 17, 92–101 10.1016/j.omtn.2019.05.007 31234009 PMC6595407

[30] Wang Y., Han C., Li M., Yu H., Duan P., Zhu H. et al. (2017) miR-613 inhibits bladder cancer proliferation and migration through targeting SphK1. J. Cell. Biochem. 9, 1213–1221 PMC5376012 28386347

[31] Jiang C., Yang Y., Yang Y., Guo L., Huang J., Liu X. et al. (2018) Long noncoding RNA (lncRNA) HOTAIR affects tumorigenesis and metastasis of non-small cell lung cancer by Upregulating miR-613. Oncol. Res. 26, 725–734 10.3727/096504017X15119467381615 29187267 PMC7844735

[32] Wang J., Yang S. and Ge W. (2018) MiR-613 suppressed the laryngeal squamous cell carcinoma progression through regulating PDK1. 119, 5118–5125 10.1002/jcb.26468 29091303

[33] Yang X., Zhang L., Song X., He W., Zhang D., Lu Q. et al. (2018) MicroRNA-613 promotes colon cancer cell proliferation, invasion and migration by targeting ATOH1. Biochem. Biophys. Res. Commun. 504, 827–833 10.1016/j.bbrc.2018.09.054 30219232

[34] Li W.T., Wang B.L., Yang C.S., Lang B.C. and Lin Y.Z. (2018) MiR-613 promotes cell proliferation and invasion in cervical cancer via targeting PTPN9. Eur. Rev. Med. Pharmacol. Sci. 22, 4107–4114 30024598 10.26355/eurrev_201807_15402

[35] Gasparri M.L. (2018) MiRNAs and their interplay with PI3K/AKT/mTOR pathway in ovarian cancer cells: a potential role in platinum resistance. J. Cell. Physiol. 144, 2313–2318 10.1007/s00432-018-2737-y PMC11813288 30109500

[36] Lu P.W., Li L., Wang F. and Gu Y.T. (2019) Inhibitory role of large intergenic noncoding RNA-ROR on tamoxifen resistance in the endocrine therapy of breast cancer by regulating the PI3K/Akt/mTOR signaling pathway. 234, 1904–1912 10.1002/jcp.27066 30145819

[37] Yang Y., Dai W., Sun Y. and Zhao Z. (2019) Long noncoding RNA linc00239 promotes malignant behaviors and chemoresistance against doxorubicin partially via activation of the PI3K/Akt/mTOR pathway in acute myeloid leukaemia cells. Oncol. Rep. 41, 2311–2320 30720129 10.3892/or.2019.6991

