# Supplementary material for: LncRNA UCA1 elevates the resistance of human leukemia cells to daunorubicin by the PI3K/AKT pathway via sponging miR-613
Source: Biosci Rep. 2021 Jun 10;41(6):BSR20201389. doi: 10.1042/BSR20201389 (PMC8193642; doi:10.1042/BSR20201389)
Supplement: Supplementary Figures S1-S3 [file BSR-2020-1389_supp.pdf]

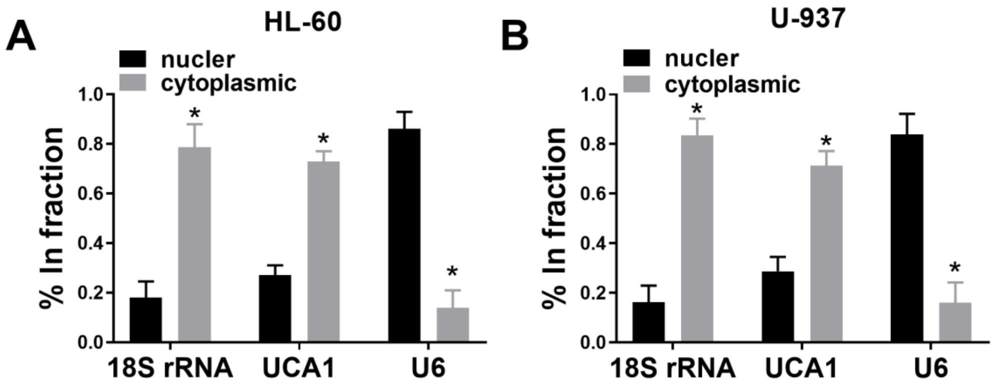

Supplementary Fig. 1 The distribution of UCA1 in HL-60 and U-937 cells. (A and B) The abundances of UCA1, 18S rRNA, and U6 in the nuclear and cytoplasmic fractions of HL-60 and U-937 cells were analyzed by qRT-PCR. *$P < 0.05$.

A

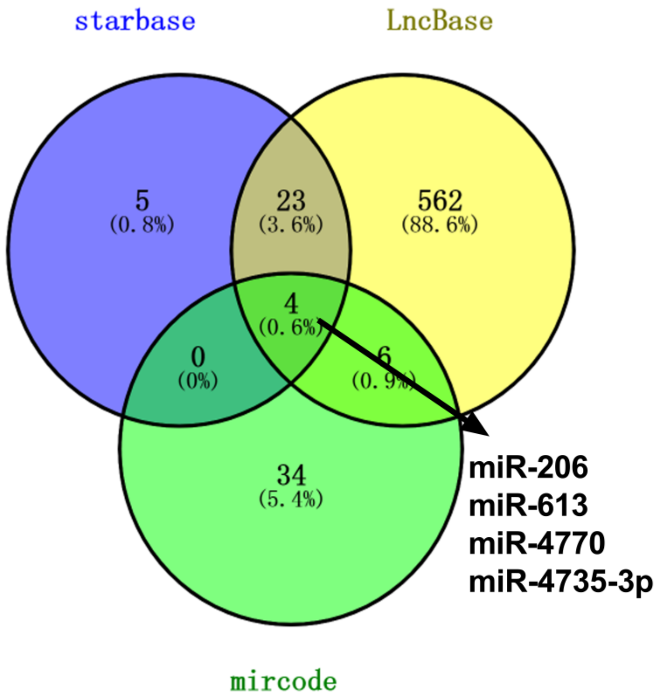

B

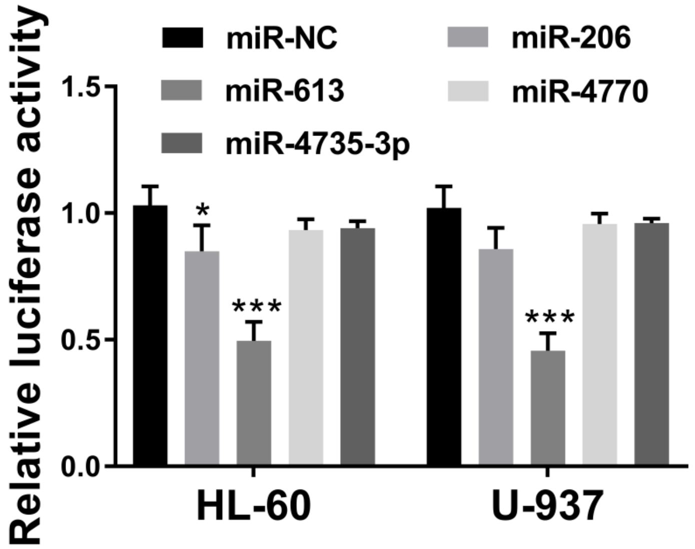

Supplementary Fig. 2 Prediction of miRNAs with UCA1 complementary sites. (A) 4 miRNAs (miR-206, miR-613, miR-4770, and miR-4735-3p) were predicted to have complementary sites to UCA1. (B) Dual-luciferase reporter assay revered the influence of miR-206, miR-613, miR-4770, and miR-4735-3p mimics on the luciferase activity of the UCA1-WT reporter in HL-60 and U-937 cells. **P* < 0.05 and ****P* < 0.001.

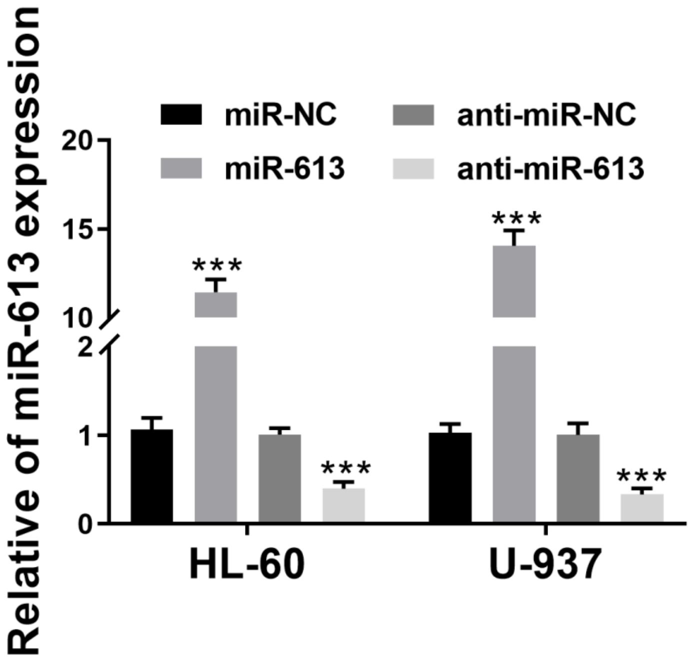

Supplementary Fig. 3 The overexpression and knockdown efficiencies of miR-613 mimic and inhibitor in HL-60 and U-937 cells were analyzed by qRT-PCR. ***$P < 0.001$.
